# Effects of the MCF-7 Exhausted Medium on hADSC Behaviour

**DOI:** 10.3390/ijms25137026

**Published:** 2024-06-27

**Authors:** Giuseppe Garroni, Sara Cruciani, Diletta Serra, Renzo Pala, Donatella Coradduzza, Maria Laura Cossu, Giorgio Carlo Ginesu, Carlo Ventura, Margherita Maioli

**Affiliations:** 1 Department of Biomedical Sciences, University of Sassari, Viale San Pietro 43/B, 07100 Sassari, Italy; giugarroni21@gmail.com (G.G.); scruciani@uniss.it (S.C.); dilettaserra9@gmail.com (D.S.); renzopala6@gmail.com (R.P.); donatella.coradduzza0@gmail.com (D.C.); 2 Department of Medical, Surgical and Experimental Sciences, University of Sassari, Viale San Pietro 8, 07100 Sassari, Italy; mlcossu@uniss.it (M.L.C.); ginesugc@uniss.it (G.C.G.); 3 National Laboratory of Molecular Biology and Stem Cell Bioengineering of the National Institute of Biostructures and Biosystems (NIBB) c/o Eldor Lab, Via Corticella 183, 40129 Bologna, Italy; carlo.ventura@unibo.it; 4 Center for Developmental Biology and Reprogramming (CEDEBIOR), Department of Biomedical Sciences, University of Sassari, Viale San Pietro 43/B, 07100 Sassari, Italy

**Keywords:** stem cells, ADSC, epigenetic mechanism, miRNA, stem cell differentiation, conditioned media

## Abstract

Stem cells possess the ability to differentiate into different lineages and the ability to self-renew, thus representing an excellent tool for regenerative medicine. They can be isolated from different tissues, including the adipose tissue. Adipose tissue and human adipose-derived stem cells (hADSCs) are privileged candidates for regenerative medicine procedures or other plastic reconstructive surgeries. The cellular environment is able to influence the fate of stem cells residing in the tissue. In a previous study, we exposed hADSCs to an exhausted medium of a breast cancer cell line (MCF-7) recovered at different days (4, 7, and 10 days). In the same paper, we inferred that the medium was able to influence the behaviour of stem cells. Considering these results, in the present study, we evaluated the expression of the major genes related to adipogenic and osteogenic differentiation. To confirm the gene expression data, oil red and alizarin red colorimetric assays were performed. Lastly, we evaluated the expression of miRNAs influencing the differentiation process and the proliferation rate, maintaining a proliferative state. The data obtained confirmed that cells exposed to the medium maintained a stem and proliferative state that could lead to a risky proliferative phenotype.

## 1. Introduction

Stem cells are cells that possess the ability to differentiate into different lineages and the capability of self-renewal. They are classified according to their regenerative potential and origin. In particular, according to their origin, they are classified into adult (within specialised tissues) or embryonic [1,2,3].

In tissues, adult stem cells, through the differentiation process, are responsible for normal cell turnover. Adult stem cells can be isolated from different tissues, such as bone marrow, the umbilical cord, skin, and adipose tissue [4,5,6].

In particular, the adipose tissue and its derived stem cells are excellent candidates for regenerative medicine procedures or other plastic reconstructive surgeries [7,8,9].

Several chemical and physical stimuli can regulate the differentiation of stem cells in vitro for their use in regenerative medicine [10,11,12,13,14].

Cell differentiation is a process that leads to a specialisation of stem cells towards a specific phenotype typical of the tissue in which the stem cells reside, thus enabling the re-establishment of tissue homeostats [15,16].

The niche plays a key role in maintaining a stemness state, or in inducing stem cell lineage commitment through the generation of chemical and physical microenvironmental signals in response to dynamic tissue requirements. Secreted signals can be autocrine, coming from the cells that make up the niche, or systemic, playing a key role in regulating stem cells’ fate. Other forms of physical signalling arising from the interaction with the extracellular matrix [ECM] through adhesion receptors, as well as physical and topological mechanical cues, can also provide stem cells with key information to regulate their final fate [17,18,19].

The molecular mechanism underlying cell differentiation is a downregulation of genes responsible for maintaining stemness in favour of genes typical of differentiating paths. Differentiation can be induced in vitro with the help of different interacting compounds, stimulating stem cells to move towards a certain lineage over another [16,20,21,22,23].

Epigenetic influences, e.g., miRNAs (small non-coding RNAs of 20–25 nucleotides), play an important role in gene regulation. They are involved in several physiological cellular processes, including stem cell differentiation, as well as pathological ones such as malignant transformations [23,24,25,26].

In a previous study, we analysed how an exhausted medium of a breast cancer line, MCF-7, influenced the behaviour of stem cells isolated from adipose tissue. The results showed that the exhausted medium was able to induce an overexpression of stemness genes. An increase in stemness genes could indicate maintenance of an undifferentiated stem cell state and increased proliferation [27]. Within this context, in a pathological environment, several factors can induce changes in stem cell behaviour by inducing a maintenance of stemness, which correlates with increased cell proliferation, often resulting in a malignant phenotype [28,29].

The tumour microenvironment, for instance, is able to influence MSCs so as to stimulate tumour growth and induce angiogenesis, immune evasion, and resistance to chemotherapy [30,31].

In this study, we analysed cell behaviour during differentiation, with particular attention to the main markers of adipogenic and osteogenic commitment and epigenetic factors. We assessed the main markers of adipogenic and osteogenic phenotypes by gene expression and colorimetric assays. We also analysed the expression of different miRNAs—hsa-miR-145-5p (miR-145), hsa-miR-148a-3p (miR-148) and hsa-miR-185-3p (miR-185)—that play a key role in the regulation of the cellular differentiation process [14,28].

## 2. Results

### 2.1. MCF-7-E.M Counteracts Adipose-Derived Mesenchymal Stem Cell (ADSC) Expression of Key Adipogenic Genes

**Figure 1:**
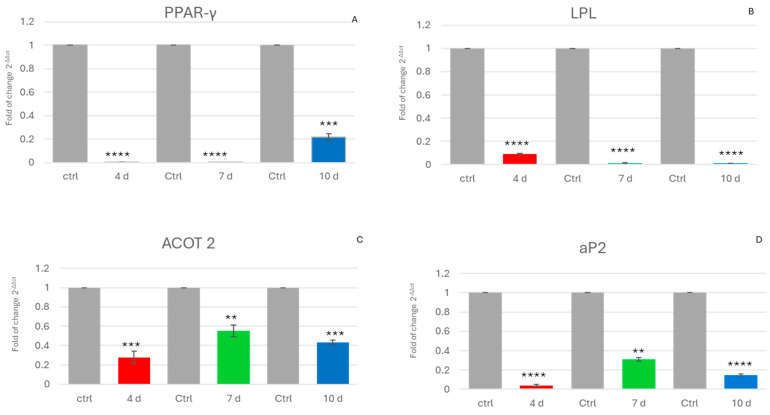
Expression of adipogenic genes: The expression of PPAR-γ (**A**), LPL (**B**), ACOT 2 (**C**), and aP2 (**D**) was evaluated in hADSCs cultured in the presence of 4d-MCF-7-E.M (4 d: red bars), 7d-MCF-7-E.M (7 d: green bars), or 10d-MCF-7-E.M (10 d: blue bars). The mRNA levels for each gene were normalised to glyceraldehyde-3-phosphate-dehydrogenase (GAPDH) and expressed as the fold change (2^−ΔΔCt^) of the mRNA levels observed in untreated control hADSCs (Ctrl: grey bars), defined as 1 (mean ± SD; *n* = 6). Data are expressed as the mean ± SD relative to the control (** *p* ≤ 0.01), (*** *p* ≤ 0.001), (**** *p* ≤ 0.0001).

Figure 1 shows the expression of genes that are usually upregulated during the adipogenic commitment of ADSCs. The expression of PPAR-γ, a main orchestrator of adipogenic commitment, was downregulated in ADSCs exposed to the MCF-7-E.M (Figure 1A). The same figure shows the effects of the exhausted medium on the gene expression of lipoprotein lipase (LPL), (Figure 1B), acyl-CoA thioesterase 2 (ACOT2) (Figure 1C), and adipocyte fatty acid-binding protein, aP2 (Figure 1D) in ADSCs. These genes were also downregulated in cells exposed to the MCF-7-E.M recovered at different timepoints.

### 2.2. In Cells Exposed to MCF-7-E.M, Gene Expression of Osteogenic Markers Is Reduced

Another effect of the exhausted medium was the downregulation of the main genes involved in osteogenic differentiation. Specifically, the expression of all of them was reduced in cells exposed to MCF-7-E.M collected at 4 d, 7 d and 10 d compared to controls exposed to growth medium alone for ADSCs.

**Figure 2:**
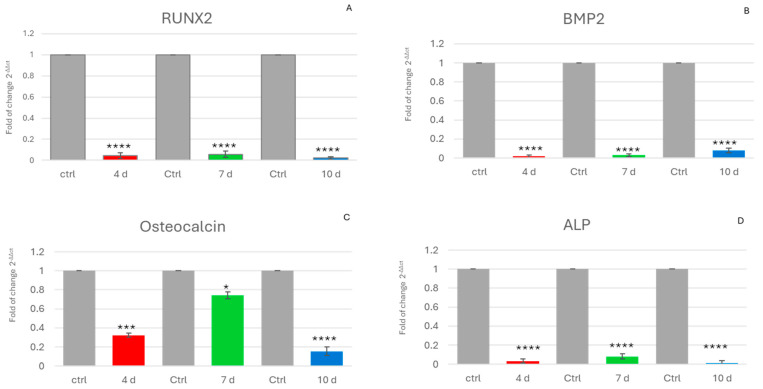
Expression of osteogenic genes: The expression of RUNX2 (**A**), BMP2 (**B**), osteocalcin (**C**), and ALP (**D**) was evaluated in hADSCs cultured in the presence of 4d-MCF-7-E.M (4 d: red bars), 7d-MCF-7-E.M (7 d: green bars), or 10d-MCF-7-E.M (10 d: blue bars). The mRNA levels for each gene were normalised to glyceraldehyde-3-phosphate-dehydrogenase (GAPDH) and expressed as the fold change (2^−ΔΔCt^) of the mRNA levels observed in untreated control hADSCs (Ctrl: grey bars), defined as 1 (mean ± SD; *n* = 6). Data are expressed as the mean ± SD relative to the control (* *p* ≤ 0.05), (*** *p* ≤ 0.001), (**** *p* ≤ 0.0001).

Figure 2 shows the gene expression data of Runt-related transcription factor 2 (RUNX2) (Figure 2A), bone morphogenetic protein 2 (BMP2) (Figure 2B), osteocalcin (OCN) (Figure 2C), and alkaline phosphatase (ALP) (Figure 2D), which play key roles in regulating osteogenic differentiation.

### 2.3. Morphological Changes in ADSCs Exposed to MCF-7-E.M

**Figure 3:**
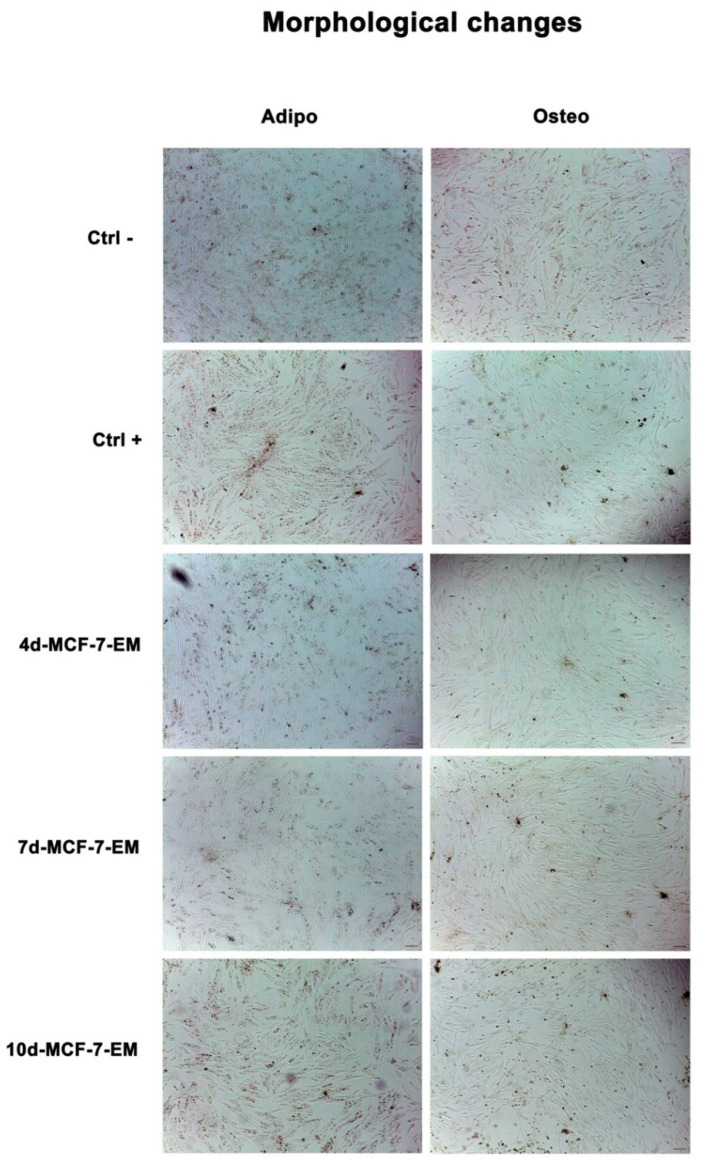
Optical microscope analysis of ADSC morphology after exposure to MCF-7-E.M figure shows morphological changes in cells exposed to 4d-, 7d-, or 10d-MCF-7-E.M after 7 days in culture, as compared to untreated control cells (Ctrl). Ctrl+ represents cells exposed to the differentiation medium alone. Scale bar = 100 µm.

To evaluate morphological changes during differentiation, ADSCs were evaluated under an optical microscope (Leica, Nussloch, Germany) after 7 days in culture, as described above. We observed significant changes in cells exposed to the differentiation medium alone (Ctrl+), especially for adipogenic differentiation, as compared to control untreated cells (Ctrl). ADSCs exposed to MCF-7-E.M appeared extremely similar to untreated control cells for 4d-MCF-7-E.M and 7d-MCF-7-E.M-treated cells, while a slight change in morphology was observable in 10d-MCF-7-E.M-treated cells, as compared to untreated controls (Figure 3).

### 2.4. Exhausted Medium Interferes with Cell Differentiation

**Figure 4:**
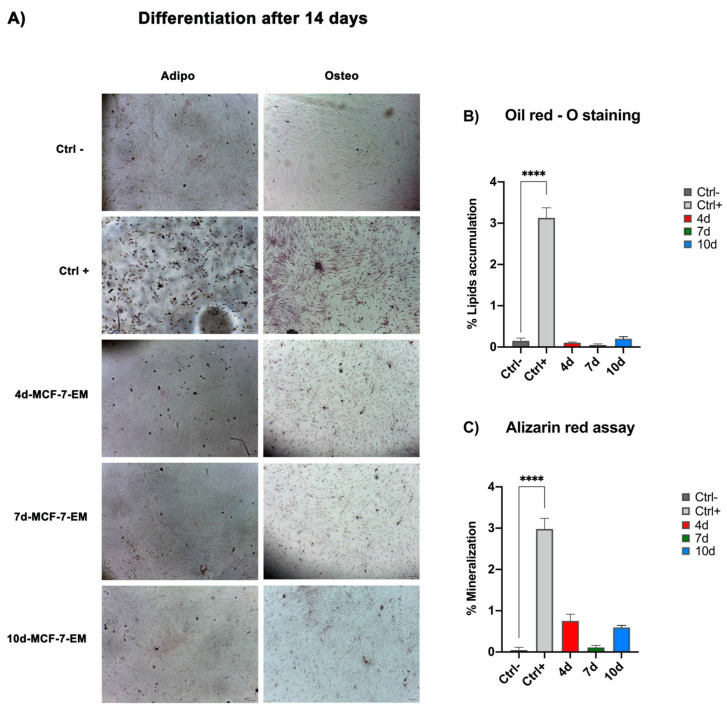
Effects of MCF-7-E.M in ADSCs during adipogenic and osteogenic differentiation: (**A**) Lipid and calcium accumulation, after 14 days of treatment, in ADSCs exposed to 4d-MCF-7-E.M (4 d: red bars), 7d-MCF-7-EM (7 d: green bars), or 10d-MCF-7-EM (10 d: blue bars). Positive controls (CTRL+: light grey bars) are ADSCs cultured in adipogenic or osteogenic conditioned medium. Scale bar = 100 μm. The percentage of lipid accumulation (**B**) or mineralisation (**C**) was calculated using ImageJ. Data are expressed as the mean ± SD and are representative of 6 different experiments. An average was made from three technical replicates (**** *p* ≤ 0.0001).

We then found that, during the adipogenic or osteogenic differentiation, ADSCs exposed to 4d-, 7d-, or 10d-MCF-7-E.M for 3 days and then maintained in the differentiation-conditioned medium showed reduced lipid accumulation or mineralisation, as compared to positive controls cultured in the presence of the differentiation medium alone (Figure 4).

### 2.5. miRNA Expression in ADSCs Exposed to MCF-7-E.M

**Figure 5:**
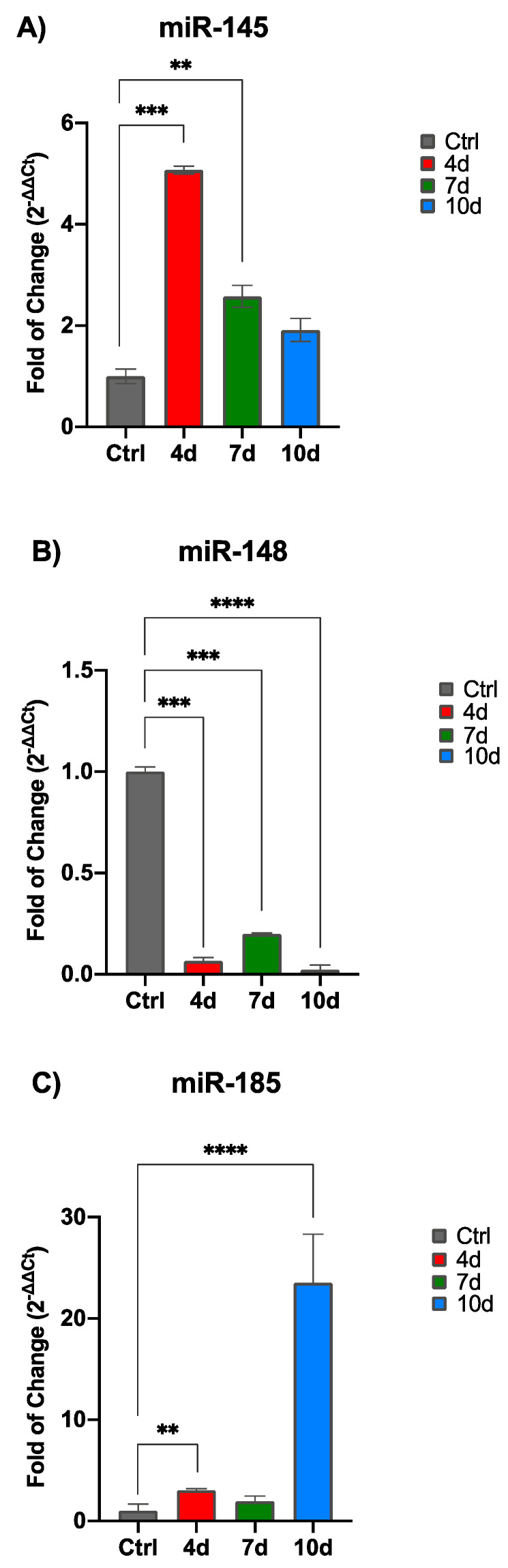
The expression of miR-145 (**A**), miR-148a (**B**), and miR-185 (**C**) was evaluated in adipose-derived stem cells (hADSCs) cultured with 4d-MCF-7-E.M (4 d: red bars), 7d-MCF-7-E.M (7 d: green bars), or 10d-MCF-7-E.M (10 d: blue bars). The mRNA levels for each gene were normalised to U6snRNA and expressed as the fold change (2^−∆∆Ct^) of the mRNA levels observed in untreated control hADSCs (Ctrl: grey bars), defined as 1 (mean ± SD; *n* = 6). Data are expressed as the mean ± SD relative to the control (** *p* ≤ 0.01), (*** *p* ≤ 0.001), (**** *p* ≤ 0.0001).

The miRNA expression was evaluated in cells exposed to MCF-7-E.M collected after 4, 7, and 10 days in culture (Figure 5); miR-145 (Figure 5A) was significantly upregulated in hADSCs exposed to MCF-7-E.M, being significantly upregulated in 4d-MCF-7-E.M-exposed hADSCs, as compared to control cells. An opposite trend could be observed for miR-148 expression (Figure 5B), being significantly downregulated in hADSCs exposed to MCF-7-E.M for all conditions, as compared to untreated control cells. Meanwhile, miR-185 (Figure 5C) was slightly upregulated in hADSCs exposed to 4d-MCF-7-E.M and 7d-MCF-7-E.M, while it was significantly upregulated in hADSCs exposed to 10d-MCF-7-E.M, as compared to control cells.

## 3. Discussion

The external environment plays a key role in directing the fate and behaviour of stem cells. External factors, such as the age of the donor, the number of passages, and the quality of the plastic surface and media (such as scaffolds, cell culture media, and their supplementary factors), oxygen concentration, and physical and chemical factors, including cytokines and other soluble factors, are all able to influence stem cell behaviour [32,33]. Adipose tissue and the stem cells contained within it are used in various areas of regenerative medicine, as well as plastic and reconstructive surgery [34,35].

A pathological environment, such as that of a tumour, can influence stem cell behaviour. Understanding how an inflammatory and factor-rich environment released by tumour cells can influence stem cell behaviour represents a major issue for the safe use of stem cells. Furthermore, this kind of investigation may help in better understanding which factors can induce certain tumour characteristics [27,28,36]. MCF-7 is one of the most widely used cell models for in vitro breast cancer research and drug testing. As an oestrogen-dependent breast cancer cell line, it has been used as a model of study for more than 40 years by various research groups. The reason for this lies in the fact that most breast cancers are oestrogen-dependent [37,38].

We previously demonstrated that the exhausted medium recovered from MCF-7 induced an overexpression of stemness genes in ADSCs [27], thus leading to the maintenance of a pluripotent state associated with increased cell proliferation [39].

To further analyse the previously observed features of these transformed stem cells, here we decided to evaluate the expression of some of the main markers of adipogenic (Figure 1) and osteogenic (Figure 2) differentiation.

As shown in Figure 1, downregulation of genes related to adipogenic differentiation, such as PPAR-γ, aP2, and LPL, was detected in cells exposed to MCF-7-E.M. These genes, like peroxisome proliferator-activated receptor γ (PPAR-γ), play a key role in determining ADSC differentiation. In particular, PPAR-γ acts as a master regulator of the induction of the adipogenic phenotype through a fine-tuning of lipogenesis, as well as adipocyte survival and function [40,41].

From our results, it is evident that the expression of this key gene is significantly reduced in cells exposed to MCF-7 exhausted medium.

These findings significantly expand our previous analysis, showing that the capability of the exhausted medium to elicit an overexpression of stemness genes [27] involved a remarkable impairment in the transcriptional machinery committed to sustaining the entire process of adipogenesis (Figure 1).

PPAR-gamma is involved in the activation of other genes engaged in differentiation, such as the adipocyte-specific aP2 gene (adipocyte fatty acid-binding protein 2) and lipoprotein lipase (LPL), another important player involved in fatty acid metabolism [42,43,44].

The MCF-7 medium recovered after 4 d, 7 d, and 10 d in culture was able to interfere with the expression of such markers of the intermediate and late adipose phenotype, ultimately impairing triglyceride accumulation, an essential hallmark of metabolic specification in differentiating ADSCs, as demonstrated by the oil red colorimetric analyses (Figure 4).

MCF-7-E.M induced an additional relevant detrimental effect, consisting of the downregulation of gene expression of the main controllers of osteogenic differentiation, such as RUNX2.

This gene is most essential for the commitment, differentiation, matrix production, and mineralisation of osteoblasts during bone formation [45,46,47]. It should be noted that the exhausted-medium-induced inhibition of RUNX2 gene expression was associated with the downregulation of a panel of key downstream genes, including osteocalcin, a bone-specific protein synthesised by osteoblasts that is an important good marker of osteogenic maturation [48,49], BMP-2 (bone morphogenetic protein-2), which is considered to be one of the most active osteogenic induction factors involved in bone tissue regeneration and has been extensively studied [50,51], and ALP (alkaline phosphatase), encoding an important enzyme in the terminal biomineralisation process [52,53,54]. On the whole, the downregulation of the expression of these genes indicates the loss of the exposed ADSCs’ ability to retain their osteogenic potential. An interference of the exhausted medium in osteogenic differentiation was further inferred from the alizarin red assay (Figure 4), which provided evidence for a remarkable reduction in calcium accumulation in exposed cells, confirming the results yielded by gene expression studies at the cellular phenotypic level.

In addition to genes correlated with adipogenic and osteogenic differentiation, we analysed the expression of miRNAs involved in these differentiation process, as well as in the maintenance of a proliferative state in cells exposed to MCF-7-E.M at 4, 7, and 10 days.

miRNAs consistently affect the differentiation process of MSCs, directly or indirectly controlling the expression of genes essential in cell lineage commitment and specification [55].

In our study, we analysed the expression of miRNAs involved in cellular mechanisms (miR-145, together with miR-185 and miR-148), such as cell proliferation, differentiation, apoptosis, and metastasis in the tumour environment [56,57,58].

Overexpression of miR-145 would promote the proliferation and migration of ADSCs. The observed increase in the levels of this miRNA in cells exposed to MCF-7-E.M (Figure 5) is consonant with an increased expression of stemness-associated marker levels [27]. Moreover, the overexpression of miR-145-5p in ADSCs has been associated with the promotion of cell proliferation, cell migration, and a reduction in cell senescence [59]. Another effect of the upregulation of miR-145 could be the maintenance of stemness at the expenses of osteogenesis, as the observations suggest that inhibition of miR-145-5p conversely increased the osteogenic potential of ADSCs [60].

Akin to these considerations, Guo et al. (2012) demonstrated that induced expression of miR-145 during differentiation could also inhibit adipogenesis [61], suggesting that the overexpression of this miRNA in MCF-7-E.M-exposed cells may have acted as a common inhibitory mechanism in both the osteogenic and adipogenic repertoire of ADSCs.

Compounding the effect of the exhausted medium on miRNA expression, MiR-148b-3p was shown to be downregulated in exposed ADSCs (Figure 5B). Its inhibition could be involved in the currently observed downregulation of the main markers of adipogenic commitment, as suggested by the observation that miR-148a inactivation in adipogenic progenitor cells attenuated adipocyte differentiation [62].

MiR-148a-3p, well known to be downregulated in MCF-7 and associated with the diagnosis of breast cancer [62], plays a crucial role in MCF-7 survival and proliferation, inhibiting apoptosis. Within this context, other authors have demonstrated an overexpression of this miRNA, being associated with increased apoptosis and reduced cell viability [63]. Here, we show (Figure 5B) that Mir-148 is downregulated in cells cultured in the presence of MCF-7 exhausted medium, and its inhibition could be involved in the downregulation of the main markers of adipogenic commitment. Moreover, miR-148a induced the activation of PPAR, an important factor in adipogenesis [64].

The last of the three miRNAs analysed, miR-185-5p (Figure 5C), was found to be overexpressed in ADSCs that had been exposed to MCF-7-E.M, with significant expression in the medium recovered at 10 days. miR-185-5p has been shown to correlate with lymph node metastasis in breast cancer [65,66].

Furthermore, as shown by Cui Q et al. in 2019, this microRNA is related to inhibition of osteogenic differentiation, since primary osteoblasts and mesenchymal stem cells derived from miR-185-knockout (KO) mice showed increased osteogenesis [67]. Chang H. et al., in 2017, demonstrated that, in MC3T3-E1 cells transfected with miR-185-5p and committed to osteogenesis, ALP activity was significantly lower, suggesting that miR-185-5p acts as a negative regulator of early osteogenesis [68].

mir-185 is also able to affect adipogenic differentiation. Within this context, other authors have demonstrated that the overexpression of miR-185 resulted in a decrease in lipid accumulation during differentiation of 3T3-L1 preadipocytes. Furthermore, knockdown of endogenous miR-185 via antagomirs promoted the differentiation of 3T3-L1 cells. In the same study, the authors indicated the sterol regulatory element-binding protein 1 (SREBP-1) gene as a probable target [69].

These effects of mir-185-5p could be correlated with the maintenance of a proliferative and dedifferentiated state of ADSCs exposed to the exhausted medium, posing a serious threat within a tissue undergoing a malignant transformation, as it occurs whenever stem cell proliferation becomes unplugged from a differentiating potential [70].

On the whole, the present findings raise a relevant cautionary note and a grim reminder that transplanting stem cells within tissues that have previously been challenged by neoplastic processes may expose the transplanted stem cells to microenvironmental cues, leading to a substantial drift from the desired cell therapy proposes. In particular, the transplantation of adipose tissue derivatives containing ADSCs has been increasingly used as a breast reconstruction strategy in patients previously affected by breast cancer and suffering from structural and functional damages induced by radiotherapy procedures. The current observations prompt further thorough studies to establish whether breast reconstruction entrusted to stem cell therapy may indeed fall within a hostile tissue milieu, turning the desired tissue rescue into uncontrolled stem cell proliferation and adipogenic impairment, with desultory aesthetic/functional outcomes and the risk of tumour relapse.

Stress induces modifications in stem cells, triggering transformations in their physiological behaviour. The evaluation of these responses in this manuscript could pave the way for future evaluations of the effects of environmental stressful conditions, such as the absence of gravity, on stem cell signatures.

## 4. Materials and Methods

### 4.1. Stem Cell Isolation and Expansion, and Experimental Design

Human Adipose tissue-derived stem cells (hADSCs) were obtained from biopsies of adult male and female patients after approval by the Human Study Review Ethics Committee of Sassari (n_ ETIC 240I/CE 26 July 2016, Ethics Committee, ASL Sassari), after signing written informed consent. Cells were isolated and cultured as previously described [20].

**Figure 6:**
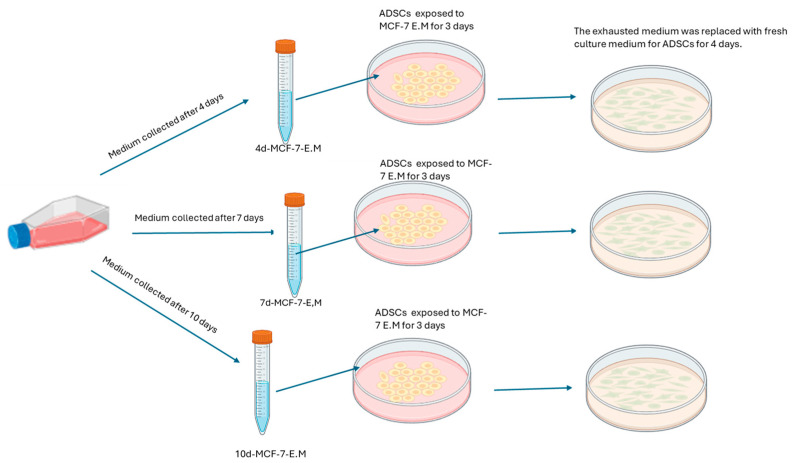
Experimental design.

The MCF-7 cell line (ATCC, Manassas, VA, USA) and ADSCs were expanded as previously described and reported in Figure 6 [27].

#### ADSC Treatment

ADSCs were seeded on 6-well plates and then exposed for 3 days to 1.5 mL of exhausted medium obtained from MCF-7 (MCF-7-E.M) cultured for 4 (4d-MCF-7-E.M), 7 (7d-MCF-7-E.M), or 10 (10d-MCF-7-E.M) days. A group of cells was exposed to 1.5 mL of basic growth medium for ADSCs, representing the cells used as untreated controls in this study, referred to as Ctrl. The 6-well cell culture plates were placed in a 37 °C incubator with 5% CO_2_ and saturated humidity. After 3 days of treatment with the MCF-7-E.M recovered at 3 different time points, the medium was removed and replaced with 1.5 mL of fresh hADSCs basic growth medium for an additional 4 days, for a total of 7 days of treatment, before proceeding with the subsequent analysis. Figure 6 shows the treatment plan for hADSCs [27].

### 4.2. RNA Extraction and Gene Expression Analysis

From ADSCs seeded on 6-well plates and then exposed as previously described by Garroni G. et al. in 2021 [27], quantitative real-time polymerase chain reaction was performed. Total mRNA was isolated from ADSCs grown under the conditions described above using the Ambion Pure Link™ RNA Mini Kit (Life technologies, Invitrogen, Carlsbad, CA, USA) according to the manufacturer’s protocol. RNA quantity and purity were measured using NanoDrop (Thermo Scientific, Waltham, MA, USA). Quantitative polymerase chain reaction (RT-qPCR) was performed using a CFX thermocycler (Bio-Rad, Hercules, CA, USA) in triplicate under standard RT-qPCR conditions (55 °C for 10 min, 95 °C for 1 min, and then cycled at 95 °C for 10 s and 60 °C for 30 s for 40–45 cycles), using the Luna **^®^** Universal One-Step RT-qPCR kit (New England Biolabs, 240 County Road, Ipswich, MA, USA). Luna Universal One-Step Reaction Mix (2×) (10 µL), 1 µL of Luna WarmStart^®^ RT Enzyme Mix (20×), 0.4 µM (0.8 µL) of each primer, and 2.5 µL (2.5 ng) of the total RNA template were mixed in 20 µL volumes and added to each reaction. Relative expression was determined using the ‘delta-CT method’, with glyceraldehyde-3-phosphate dehydrogenase (GAPDH) as the reference gene. The mRNA levels of the treated cells were expressed as the variation (2^−∆∆Ct^) in the mRNA levels observed in the untreated control cells. RT-qPCR analysis was performed for the following set of genes: for adipogenesis, peroxisome proliferator-activated receptor gamma (PPAR-γ), adipocyte protein 2 (aP2), lipoprotein lipase (LPL), and acyl-CoA thioesterase 2 (ACOT2); for osteogenesis, Runt-related transcription factor 2 (RUNX2), bone morphogenetic protein 2 (BMP2), osteocalcin (OCN), and alkaline phosphatase (ALP). All primers used (Thermo Fisher Scientific, Waltham, MA, USA) are described in Table 1.

### 4.3. Extraction and Expression of miRNAs

The expression levels of the miRNAs analysed (Table 2) were assessed by reverse transcription followed by polymerase chain reaction (RT-PCR) using the TaqMan^®^ MicroRNA Reverse Transcription Kit (Thermo Fisher Scientific, Grand Island, NY, USA), as previously described by Balzano et al. in 2020 [28].

Total RNA was extracted using the Mirvana MIRNA ISO Kit 10-40ISO (Life Technologies, Carlsbad, CA, USA) according to the manufacturer’s protocol, with a final elution volume of 15 μL.

The raw Ct values for each miRNA and U6snRNA were checked for normal distribution. The Kruskal–Wallis test and the Wilcoxon signed-rank test were applied to compare the groups at different observation times, assuming a *p*-value < 0.05 as statistically significant. All analyses and graphs were performed and constructed with GraphPad Prism 9.0 software (GraphPad, San Diego, CA, USA).

The identifying sequences and symbols were retrieved from miRBase and are shown in Table 2.

### 4.4. Colorimetric Assays to Assess Stem Cells’ Differentiation

To confirm gene expression data by real-time PCR, the oil red and alizarin red colour assays were performed. ADSCs were grown on 24-well plates. Once confluency was reached, the cells were exposed to treatment with 4d-MCF-7-E.M, 7d-MCF-7-E.M, or 10d-MCF-7-E.M, as previously described [27], while a group of cells was maintained in culture with only growth medium for ADSCs. After treatment with the different exhausted media, the cells were exposed to adipogenic (StemPro™ Adipocyte Differentiation Kit (TRT) medium (Gibco Life Technologies, Grand Island, NY, USA)) and osteogenic differentiation media (StemPro™ Osteogenesis Differentiation Kit (TRT) medium (Gibco Life Technologies, Grand Island, NY, USA)) for 14 days, while one group, a positive control of differentiation, was exposed to the lineage-specific differentiation medium alone, as previously described. Another group of cells was used as a negative control using growth medium for ADSCs alone. Subsequently, the oil red and alizarin red tests were performed.

#### 4.4.1. Oil Red Staining

To assess the accumulation of lipid droplets, ADSCs were cultured for 14 days under the above-described conditions. After 14 days, the cells were fixed for 30 min at room temperature in 10% formalin, and then washed twice in H_2_O and 60% isopropanol for 5 min. The cells were then incubated in oil red solution (Oil Red O Solution (Sigma-Aldrich, St. Louis, MO, USA)) for 15 min at room temperature and washed once in 60% isopropanol and three times in H_2_O to remove excess solution. Adipogenesis was assessed by light microscopy, and lipid accumulation analysis was performed using ImageJ image analysis software version 1.8.0 (ImageJ, National Institutes of Health, United States Department of Health and Human Services, Bethesda, MD, USA).

#### 4.4.2. Alizarin Red Assay

To assess calcium deposition within the cells, the alizarin red assay was performed.

Cells were cultured for 14 days on 24-well tissue culture plates (BD-falcon, BD Biosciences, Bedford, MA, USA), in the presence of osteogenic differentiation medium or basic culture medium (CTRL). After 14 days, the cells were fixed with 10% formalin for 15 min at room temperature, washed three times in distilled water (ddH_2_O), and then stained with 2% alizarin red S solution (Santa Cruz Biotechnology, Dallas, TX, USA) for 20 min at room temperature. The cells were carefully washed three times in ddH2O and observed under a light microscope to analyse calcium deposition.

Calcium deposition analysis was performed using ImageJ image analysis software, version 1.8.0 (ImageJ, National Institutes of Health, United States Department of Health and Human Services, Bethesda, MD, USA).

### 4.5. Statistical Analysis

GraphPad Prism 9.0 software (GraphPad, San Diego, CA, USA) was used to perform the statistical analyses. The experiments were performed twice, with three technical replications for each treatment. The Kruskal–Wallis rank-sum test, two-way ANOVA with Tukey correction, and Wilcoxon signed-rank test were used for this study, assuming a *p*-value < 0.05 as statistically significant; (* *p* < 0.05), (** *p* ≤ 0.01), (*** *p* ≤ 0.001), (**** *p* ≤ 0.0001).

## 5. Conclusions

On the whole, we provide evidence that, within an oncogenic environment, such as that brought about by MCF-7-E.M, ADSCs, which form a major component of scaffolding and tissue homeostasis in the breast tissue, may themselves become tumorigenic elements, losing their ability to differentiate, and supporting the growth and progression of locally developing tumours.

## Figures and Tables

**Table 1:** Primer sequences.

Name	Reverse	Forward
PPAR-γ	GTGAAGACCAGCCTCTTTGC	AATCCGTCTTCATCCACAGG
aP2	TCATTTTCCCACTCCAGCCC	AGACATTCTACGGGCAGCAC
LPL	GGGACCCTCTGGTGAATGTG	CAGGATGTGGCCCGGTTTAT
ACOT2	TCTTGGCCTCGAATGGTATC	GAGGTCTTCACACTGCACCA
RUNX2	TCGTCCACTCCGGCCCACAA	CTGTGCTCGGTGCTGCCCTC
BMP2	GGCTGACCTGAGTGCCTGCG	GCGTTGCTGCTTCCCCAGGT
OCN	GACACCCTAGACCGGGCCGT	GAGCCCCAGTCCCCTACCCG
ALP	GCATTGGTGTTGTACGTCTTG	CAACCCTGGGGAGGAGAC
GAPDH	GACAAGCTTCCCGTTCTCAG	GAGTCAACGGATTTGGTCGT

**Table 2:** Identifying sequences and symbols of miRNAs.

Accession ID Number	Symbol	Sequence
MIMAT0000437	hsa-miR-145-5p	GUCCAGUUUUCCCAGGAAUCCCU
MIMAT0000243	hsa-miR-148a-3p	UCAGUGCACUACAGAACUUUGU
MIMAT0004611	hsa-miR-185-3p	AGGGGCUGGCUUUCCUCUGGUC

## Data Availability

Data are contained within the article.
